# Physiological response and phytoremediation potential of dioecious *Hippophae rhamnoides* inoculated with arbuscular mycorrhizal fungi to Pb and Zn pollution

**DOI:** 10.3389/fpls.2023.1321885

**Published:** 2024-01-03

**Authors:** Ling Fang, Zhenxiong Zeng, Qi Jia, Yuhu Lin, Hao Chen, Yunxiao He, Juan Chen

**Affiliations:** Engineering Research Center of Chuanxibei Rural Human Settlement (RHS) Construction at Mianyang Teachers’ College of Sichuan Province, Mianyang Teachers’ College, Mianyang, China

**Keywords:** sexual dimorphism, *Glomus mosseae*, heavy metal stress, mycorrhiza effect, photosynthesis, antioxidant enzymes

## Abstract

Plant-microorganism combined remediation of heavy metal pollution has been reported, but little attention has been paid to the effect of arbuscular mycorrhizal (AM) fungi on phytoremediation of dioecious plants under heavy metal pollution. In this study, the growth, physiological responses and phytoremediation traits of *Hippophae rhamnoides* were determined to evaluate whether sex-specific ecophysiological responses and phytoremediation capacities of females and males are affected by additional AM fungi (*Glomus mosseae*) under heavy metal treatments. The results showed that excess Pb and Zn stresses inhibited photosynthetic capacities of both sexes. However, inoculated AM fungi treatment increased the activity of photosynthesis, content of photosynthetic pigment, activity of superoxide dismutase, the content of proline and root Pb content and enrichment coefficient of males while decreased root Pb content of females under Pb stress. On the other hand, inoculated AM fungi treatment increased the photosynthetic activities and Pro accumulation of females, and activity of superoxide dismutase and transport coefficient of males under Zn stress. These results demonstrate that *H. rhamnoides* inoculated AM fungi showed significant sex-specific responses on the growth, physiological traits and phytoremediation potential to Pb and Zn stress. AM fungi significantly improved the tolerance of males to Pb stress and both sexes to Zn stress, which indicates *H. rhamnoides* and AM fungi can be used as a plant-microbial combined remediation method for Pb and Zn contaminated soil. More attention should be paid on sexual-specific responses and phytoremediation of dioecious plants to heavy metals in the future.

## Introduction

Phytoremediation technology has become the main means to solve heavy metal pollution due to its advantage of environmental protection, low cost and low risk of pollution. On the one hand, plants can reduce the migration or bioavailability of heavy metals in soil through the accumulation, chelation or mechanical stabilization of roots. On the other hand, plants can also absorb, transport and enrich heavy metals, which can reduce the concentration of heavy metals in soil to a certain extent. Excessive heavy metals in soils interfere with the absorption of nutrients required for plant growth and development, inhibit the growth of plants and symbiotic microorganisms, and change the physical and chemical properties of soils (Miransari, 2016; Wang et al., 2016). Lead (Pb), reported as the second most hazardous metal based on frequency of occurrence, toxicity and human exposure (Jennrich, 2013). Zinc (Zn) is one of the essential trace elements for the growth of plants, while excessive zinc has a negative effect on the growth and development of plants, inhibiting seed germination, root growth and photosynthetic rate, as well as leading to cell inactivation (Michael and Krishnaswamy, 2011). Plants can accumulate a certain amount of Pb in various organs in the body, but severe Pb pollution will lead to plant poisoning, inhibition of plant growth, photosynthesis and mineral absorption, as well as affect membrane structure and permeability (Emamverdian et al., 2015; Jayasri and Suthindhiran, 2017). Excessive Pb and Zn can produce a large number of reactive oxygen species (ROS) such as O^2-^ and H_2_O_2_, and induce damage to metabolic processes such as antioxidant defense systems or photosynthetic electron transport (Tanhan et al., 2007; Liao et al., 2022). Plant responses to Pb and Zn stress include various defense strategies, such as amino acid biosynthesis, especially proline (Pro) (Rucińska-Sobkowiak et al., 2013), which can be used as a metal chelating agent and protein stabilizer to reduce metal toxicity (Lamhamdi et al., 2013; Liao et al., 2022). Heavy metal stresses can activate the antioxidant defense system of plants, resulting in an increase in the activity of enzymatic antioxidants such as peroxidase (POD) and superoxide dismutase (SOD). In addition, plant cells also have non-enzymatic antioxidants to combat reactive oxygen species induced oxidative damage, including carotenoids, glutathione, phenols, flavonoids, and primary and secondary metabolites (Castañeda-Ovando et al., 2009; Hernández et al., 2009).

Microorganisms mainly participate in the process of material circulation and energy flow in the ecosystem through activities of symbiosis and decomposition, which have an impact on the composition and structural changes of aboveground vegetation communities. Such as mycorrhizal fungi and plant symbiosis and soil microbial decomposition of organic matter (Cang et al., 1988; Nanjundappa et al., 2019). Symbiotic fungi obtain carbohydrates necessary for growth and development from plants, while fungi expanding the absorption range of roots and enhancing the absorption of water and nutrients by plants (Wipf et al., 2019). Arbuscular mycorrhizal (AM) fungi, which can form symbiotic relationships with most terrestrial plants, is a class of soil fungi widely found in natural and agricultural ecosystems (Zhang et al., 2003; Ortega-Larrocea et al., 2010). Bradley et al. (1981) found that the plants that survived well in heavy metal mining areas were mostly mycorrhizal plants. AM fungi can reduce the damage caused by free radicals by increasing antioxidant response, chelating heavy metals and stimulating protein synthesis genes, thereby helping plants to carry out phytoremediation (Riaz et al., 2020). AM fungi, as a kind of important endogenous mycorrhizal fungi, have strong ability to complex heavy metal elements, which can significantly improve the tolerance of host plants to excess heavy metal ions (Enkhtuya et al., 2000). AM fungi can strengthen the function of host plants to absorb and transport heavy metals (Audet and Charest, 2007). Xiao et al. (2021) proved that AM fungi can enhance the activity of acid phosphatase and antioxidant enzymes, improve their ability to scavenge ROS, and alleviate the toxicity of heavy metals through dilution, thereby reducing the negative effects of plant growth. Some studies have shown that inoculation of AM fungi can significantly improve the ability of plants to accumulate and transfer Pb or Zn, and promote the accumulation of nutrients in roots and growth of plants in heavy metal polluted soil (Gonzalez-Chavez et al., 2002; Bahraminia et al., 2016; Meyer et al., 2017). The accumulation and transfer of excessive heavy metals in soil by plants and the improvement of plant tolerance by AM fungi would imply phytoremediation potential as well as can be conducive to promote vegetation restoration in heavy metal contaminated areas.

There are about 15600 species of dioecious plants in the world, which are important components of terrestrial ecosystems (Renner, 2014). According to the theory of reproductive cost, in the case of limited resources, females allocate more resources for reproduction and defense, while males allocate more resources for growth (Stehlik et al., 2008; Lu et al., 2018). Different resource allocation strategies lead to differences in morphology, physiology and tolerance between the two sexes (Xu et al., 2010; Yang et al., 2015). And the interaction with AM fungi may affect the sexual difference between the sexes. The sexual differences in the response of dioecious plants after inoculation with AM fungi have been reported. The sexes in *Antennaria dioica* and their associated fungi respond differently to increasing temperature (Vega-Frutis et al., 2014). Significant gender differences of *Populus tomentosa* induced by AM fungi colonization were obtained in SOD and GPx activities and N and P concentrations under salt stress (Lu et al., 2014). In addition, Females of *Populus cathayana* gaining more symbiosis-mediated benefits, while the drought tolerance of male is improved (Li et al., 2015) and differences in the genders existed in growth, photosynthesis and antioxidant systems of *P. cathayana* inoculated AM fungi under salt stress (Wu et al., 2016). Therefore, it is necessary to analyze the gender differences in abiotic stress tolerance of different dioecious species and AM interactions.

*Hippophae rhamnoides* L., a dioecious deciduous shrub widely distributed in parts of Asia and Europe, can maintain normal growth under drought stress, low temperature and salt and alkali stresses (Li et al., 2005; Chen et al., 2009; Xu et al., 2009; Zhang et al., 2021). At present, the combined effects of dioecious plant species and microorganisms are less considered and the response characteristics of *H. rhamnoides* males and females after inoculation of AM fungi under heavy metal pollution are unclear. Elucidating the gender differences in dioecious *H. rhamnoides* will help to explore more effective planting patterns for heavy metal pollution remediation, and the study of *H. rhamnoides*-AM fungi combined remediation will help to further enrich bioremediation technology. We hypothesized that inoculation of AM fungi will affect the growth traits and physiological processes of *H. rhamnoides* males and females under Pb stress and Zn stress, and cause differences accumulation, transport and tolerance ability between sexes. Our study aims to answer the following question: (i) Whether the interaction between Pb or Zn stress and inoculation with AM fungi affects the sexual dimorphism of *H. rhamnoides*, and *H. rhamnoides* males and females show differences in growth performance, photosynthetic rate, antioxidant enzymes activity, osmotic adjustment substances and phytoremediation related parameters. (ii) Whether there exist differences in tolerance between sexes inoculation with AM fungi under Pb and Zn stress. It would elucidate the sexual differences of physio-biochemical responses and phytoremediation potential of dioecious *H. rhamnoides* in heavy metal pollution environment.

## Materials and methods

### Plant material and experimental design

Male and female seedlings of *H. rhamnoides* were collected from Liaoning Fuxin *H. rhamnoides* seedling cultivation base. On 30 April 2022, the seedlings were transplanted into 6L plastic pots filled with sterilized soil of the experimental site and grown in a transparent awning at engineering research center of Chuanxibei RHS construction at Mianyang Teachers’College of Sichuan Province (31°29′58″ N, 104°47′6″ E, mean elevation 490 m). A transparent canopy was set up to prevent the loss of heavy metals in the soil due to rainwater. The soil, taken from the experimental base, was dried, ground, and sieved by 2 mm sieve, then sterilized twice at 121°C for 2 h in order to remove the differences of soil microorganisms and the effect of AM fungi that may exist in the original soil. The basic physical and chemical properties of soil: pH 6.72, organic matter 43.97 g/kg, total N 0.2 g/kg, total P 0.55 g/kg, available P 13.13 mg/kg, available N 4.53 mg/kg. The inoculant was *Glomus mosseae* provided by Bank of Glomeromycota in China (BGC), with about 50 spores per gram.

The experimental layout was completely randomized with three factors (AM, sex and heavy metal). Two inoculation status (inoculated or not inoculated, +AM and -AM), two sex (Male and Female, M and F), three heavy metal treatments (0, 1000 mg Pb dry soil, 500 mg Zn dry soil). Eight replicates per treatment were included in the experiment. On 30 April 2022, 132 healthy seedlings (66 females and 66 males) of *H. rhamnoides* were chosen and transplanted into 6 L plastic pots (one seedlings per pot) filled with 2.5 Kg homogenized soil and 10 g *Glomus mosseae*. After 6 weeks of growth, the plants were subjected to Pb and Zn stress for 9 weeks. In the treatment, Pb was applied into soil by evenly adding 100mL of 12.066 mmol/L (CH_3_COO)_2_Pb·3H_2_O solution to the pots every day during the first 10 days of the treatment. The final Pb level reached 1000 mg Pb kg^-1^ dry soil. In the Zn treatment, 100 ml of deionized water containing 19.12 mmol/L ZnSO_4_·7H_2_O was evenly added to the pots every day during the first 10 days of the treatment until the final Zn level reached 500 mg Zn kg^-1^ dry soil, while control plants were given equal quantities of deionized water (Chen et al., 2011). The Pb and Zn concentrations used in this study were chosen according to the Pb and Zn content of soil around the neighboring Pb-Zn mines. The temperature and moisture conditions were consistent in each treatment during the experiment. The treatments started on 20 June 2022, and the plants were harvested on 28 August 2022.

### Determination of AM fungal infection rate

After harvest, the roots were gently washed, cut into 1cm fragment with consistent thickness and fixed in FAA (formalin: acetic acid: alcohol) in the ratio of 13:5:100 (v/v/v). The root samples were bathed in 10% KOH at 90°C for 1 hour, and washed with water after cooling until the water changed from yellow to transparent. The root segments were softened with alkaline hydrogen peroxide (3 ml NaOH + 30 ml 10% H_2_O_2_ + H_2_O to 600 ml) for 20 min, stained with 95% white vinegar + 5% blue ink, and rinsed with 3% vinegar + water after boiling water bath for 3 minutes. The stained root fragments were arranged on the slide and examined with microscope (Giovannetti and Mosse, 1980). The roots and AM fungal hyphae of *H. rhamnoides* were observed under a microscope, and 120 root segments were observed in each treatment. The infection rate: The number of observed infected root segments divided by the total number of observed root segments multiplied by 100%.

### Determination of growth performance

The plant height and basal diameter of the plant were measured the day before the application of heavy metals and the collection of samples to calculate the net increase. At the end of the experiment, four seedlings from each treatment were used for the biomass measurements. The seedlings were harvested and separated into leaves, stems and roots, and separately oven-dried at 70°C for 48 h to constant weight and weighed. Total dry matter weight equals the sum of leaf, stem and root dry matter weight. Root-shoot ratio is equal to the ratio of root to shoot dry mass.

### Gas exchange and photosynthetic pigment measurements

The net photosynthetic rate (P_n_), transpiration rate (T), intercellular CO_2_ concentration (C_i_) and stomatal conductance (G_s_) were measured by Li-6400 photosynthetic apparatus (Li-Cor, Lincoln, NE, USA), and the fifth fully expanded leaves in four randomly chosen individuals in each treatment were selected between 9: 00 a.m. and 11: 30 a.m. from August 16 to 23. The instant water use efficiency was calculated by: WUE_i_= P_n_/T. The instrument parameters were set as follows: atmospheric carbon dioxide concentration (415 ± 5) mmol/mol, air flow rate in the sample chamber of 500 μmol/s, and light intensity of 1600 μmol/(m^-2^s). Chlorophyll and carotenoids content was determined by spectrophotometry according to the method of Lichtenthaler (1987) using a UV-330 spectrophotometer (Unicam, Cambridge, UK). The total chlorophyll content (TChl) was the sum of chlorophyll a and b.

### Determination of malondialdehyde, enzyme assays and proline

MDA was determined by thiobarbituric acid method: 10% trichloroacetic acid (TCA) and grinding were used under ice bath, and the supernatant was added to 0.6% thiobarbituric acid solution for boiling water bath for 15 minutes. Then the absorbance at 450, 532 and 600 nm was determined in accordance with the method of Yin et al. (2005). The calculation formula is as follows: $\text{MDA(μmol/g FW) = }\frac{[6\text{.45(A532}-A600)-0.56A450] Vt}{{\;}^{\text{Vs}}*\text{FW}}$ . In formula, Vt: total volume of extract (ml); Vs: the volume of the extract that used for determination (ml); FW: fresh weight of sample (g).

Four individuals were selected from each treatment and 0.1 g fresh leaves were weighed separately. SOD was extracted by dry ice grinding with extraction buffer (contained 5mmol/L dithiothreitol and 5% polyvinylpyrrilidone), and POD was extracted by KH_2_PO_4_. The content of SOD was measured using a nitrogen blue tetrazolium (NBT) reduction test, and POD activity were determined by guaiacol method (Zhao et al., 2022).

Extraction of proline: weight 0.1g fresh leaves and add sulfosalicylic acid solution to grind, extract in a boiling water bath for 10 minutes, centrifuge at 3000r/min for 10 minutes after cooling, and take the supernatant for testing. Pro was measured according to the acid ninhydrin method (Silva and Matos, 2016).

### Determination of starch and soluble sugar contents

Anthrone was used to determine the content of soluble sugar and starch. Weighed 0.1 g dry samples of plant roots, stems and leaves respectively, ground with 5 ml of 80% alcohol in a mortar, and using water bath (80°C) for 40 minutes. After cooling and centrifugation, the residue was extracted multiple times and combined the supernatant. Then, add 0.5 g activated carbon to the supernatant and decolored in 80°C water bath for 30 minutes. The solution was diluted to 50 ml and filtered to determine the soluble sugar content. 3 ml of distilled water was added to the precipitate and boiled for 15 minutes and 2 ml of 9.2 mmol/L perchloric acid was added after cooling. Then, after 15 minutes of boiling water extraction, the supernatant was collected by centrifugation. The residue was added with 4.6 mmol/L perchloric acid for extraction. The supernatant was combined and diluted to 50 ml to determine the starch content.

### Determination of Pb and Zn contents, transfer coefficient and enrichment coefficient

Three replicates were selected for each treatment, and 0.2 g of dried root, stem and leaf samples were weighed and digested with nitric acid: perchloric acid (volume ratio: 5: 1) on an electric furnace controlled by a transformer (Wang, 2015). The content of heavy metal Pb and Zn in the sample was determined by inductively coupled plasma emission spectrometer (ICP-OES) after 5 ml was drawn to 25 ml volumetric flask. The Translocation coefficient was the ratio of stem and leaf metal concentration to root metal concentration (Baker et al., 1994). The enrichment coefficient is the ratio of heavy metal concentration in plants to that in soil (Tanhan et al., 2007).

### Statistical analysis

SPSS 19.0 software was used to analyze the data. One-way and three-way analyses of variance (ANOVAs) were used to analyze the difference between the different treatments (Duncan’s test). The vegan package of RStudio was used for principal component analysis (PCA) to determine the main physiological and biochemical response parameters and analyze the contribution rate of principal components in the physiological response characteristic parameters of male and female of *H. rhamnoides*. The histogram was drawn with Origin2021 software.

## Results

### Effects of heavy metal stress on the infection rate of AM fungi

The infection rate of *H. rhamnoides* showed sex-specific response under Pb and Zn treatments. Compared with the controls, the infection rate of females was significantly increased and higher than did males under Pb stress (**Table 1**). However, the infection rate of females was significantly decreased and lower than did males under Zn stress. The infection rate of males under Pb stress was significantly lower than did males under Zn stress.

**Table 1:** Growth traits of *H. rhamnoides* under Pb and Zn stresses inoculated AM fungi.

Treatment	AM infection rate (%)	Net increment of plant height(cm)	Net increment of base diameter(mm)	R/S
CF	–	13.59 ± 4.03c	1.39 ± 0.34ab	0.27 ± 0.04ab
CM	–	26.04 ± 4.75abc	1.35 ± 0.26ab	0.22 ± 0.02b
CFA	56.67 ± 3.12b	15.7 ± 2.6bc	1.31 ± 0.12ab	0.21 ± 0.02b
CMA	67.50 ± 8.78ab	23.06 ± 4.89abc	1.38 ± 0.27ab	0.2 ± 0.01b
Pb F	–	13.5 ± 3.31c	0.38 ± 0.14c	0.27 ± 0.03ab
Pb M	–	26.65 ± 3.53ab	0.53 ± 0.13bc	0.2 ± 0.01b
Pb FA	79.82 ± 2.42a	16.72 ± 4.27abc	0.44 ± 0.03c	0.21 ± 0.01b
Pb MA	60.37 ± 2.95b	29.19 ± 0.8a	0.72 ± 0.11abc	0.18 ± 0.02b
Zn F	–	19.54 ± 4.5abc	1.55 ± 0.47a	0.29 ± 0.01ab
Zn M	–	19.95 ± 4.52abc	0.73 ± 0.23abc	0.22 ± 0.04b
Zn FA	30.77 ± 7.00c	25.68 ± 3.96abc	1.45 ± 0.42a	0.35 ± 0.05a
Zn MA	82.22 ± 2.58a	25.76 ± 1.79abc	1.41 ± 0.25a	0.29 ± 0.09ab
*S*		***	ns	*
*H*		ns	***	*
*I*		ns	ns	ns
*S×H*		ns	ns	ns
*S×I*		ns	ns	ns
*I×H*		ns	ns	ns
*S×H×I*		ns	ns	ns

Different letters indicate significant differences of mean value among all treatments according to Duncan’s test (P< 0.05). Each value is the mean ± SE (n = 4). CF, CFA, CM and CMA respectively indicate females, females+AM, males, males+AM in control conditon, PbF, PbFA, PbM and PbMA respectively indicate females, females+AM, males, males+AM in Pb stress. ZnF, ZnFA, ZnM and ZnMA respectively indicate females, females+AM, males, males+AM in Zn stress. S, sex effect; I, inoculation effect; H, heavy metal effect; S×H, sex and heavy metal effect; S×I, sex and inoculation effect; I×H, inoculation and heavy metal effect; S×H×I, sex, heavy metal and inoculation effect; ns, no significant difference; *P<0.05; **0.01≤P<0.001; and ***P ≤ 0.001.

### Effects of heavy metal and AM fungi on growth and biomass allocation

It indicated that Pb stress expanded the sexual difference in the growth of *H. rhamnoides*. The net increment of plant height (NH) of females was significantly higher than that of males under the control and heavy metal treatments (**Table 1**). Compared with the controls, the net increment of base diameter (BD) of females was significantly reduced by 72.67% and 68.35% respectively under Pb and Pb+AM treatments, while no significant effect on BD was observed in Zn treatment. Meanwhile, BD of females under Zn stress was higher than females under Pb stress. The R/S of females significantly increased by 66.67% under Zn+AM treatment compared with the control and Pb treatments, and the R/S between sexes had no significantly different under each treatment.

**Figure 1:**
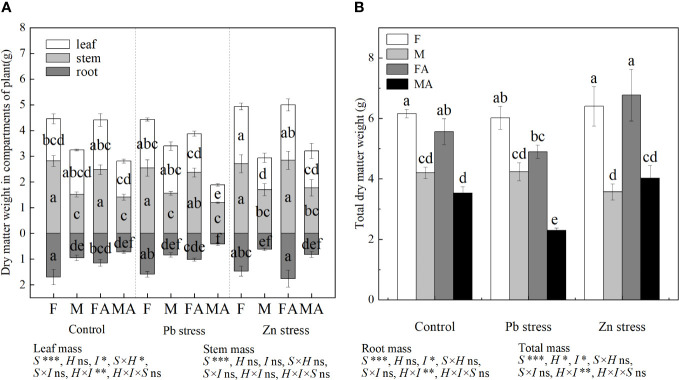
Biomass accumulation and distribution of *H. rhamnoides* under Pb and Zn stresses inoculated AM fungi: **(A)** Dry matter weight in different parts of plants, **(B)** Total dry matter weight. Different letters indicate significant differences among treatments according to Duncan’s test (P< 0.05). Each value is the mean ± SE (n = 4). F, Female; M, Male; FA, Female inoculated with AM fungi (Female + AM); MA, Male inoculated with AM fungi (Male + AM). S, sex effect; I, inoculation effect; H, heavy metal effect; S×H, sex and heavy metal effect; S×I, sex and inoculation effect; I×H, inoculation and heavy metal effect; S×H×I, sex, heavy metal and inoculation effect; ns, no significant difference; *P<0.05; **0.01≤P<0.001; and ***P ≤ 0.001.

In control conditions, no significant sexual difference was observed in leaf biomass of both sexes. Compared with the controls, the leaf biomass of females increased significantly under Zn stress (**Figure 1A**). The Pb+AM treatment expanded the sexual difference in leaf biomass of both sexes, as shown in higher leaf biomass of females than that of males. At the same time, whether or not AM fungi were inoculated, compared with controls, the sexual difference of females was significantly higher than males under Zn stress (**Figure 1A**). The stem biomass of females under the control, Pb and Zn treatments were higher than that of males. Compared with the control and Pb treatments, Zn+AM treatment significantly increased the root biomass of females. The root biomass of females under Pb+AM and Zn+AM treatments were significantly higher than males. Moreover, the male total biomass was significantly reduced under Pb+AM stress compared with the control and Zn treatments (**Figure 1B**). The statistical analysis showed that the interaction of S×H significantly affected leaf biomass, and H×I significantly affected leaf, root and total biomass (**Figure 1**).

### Effects of heavy metals and AM fungi on photosynthesis parameters

In controls, the Gs of male reduced by 42.50% while the WUEi increased by 56.10% in males after AM treatment (**Table 2**). Compared with the controls, Pb stress increased the Pn of both sexes, and Zn stress increased the Pn of females significantly. Pb stress significantly reduced the Gs, Ci and T of males, and the Ci of females compared with the controls by 65%, 35.24%, 48.73% and 16.05% respectively. Pb+AM treatment significantly increased Ci and T of both sexes, and Gs of females compared with Pb stress. Pn of females increased by 43.74% under Zn stress, while the Gs of females and males decreased by 33.33% and 45.00% respectively compared with the controls. The Gs and T of both sexes and the Ci of females under Zn+AM stress significantly increased compared with Zn stress. The Gs, Ci and T of males under Zn stress were significantly higher than those under Pb stress, and the Gs of females and the T of both sexes under Zn+AM treatment were significantly higher than those under Pb treatments. Meanwhile, compared with the controls, the WUEi of both sexes significantly increased under Pb and Zn stress, but the WUEi of female and male decreased by 44.97% and 38.01% significantly under Pb+AM treatment, and Zn+AM treatment decreased the WUEi of females significantly by 53.37%. In addition, the WUEi of males under Zn and Zn+AM treatments were lower than that under Pb and Pb+AM treatment. In short, under Pb or Zn treatments, the photosynthetic traits of *H. rhamnoides* showed a decrease as a whole respectively, while the photosynthetic indexes were improved to a certain extent after inoculation with AM fungi, and the promotion effect of Zn+AM treatment on photosynthesis was higher than that of Pb+AM treatment. The interaction of S×H and S×I significantly affected Gs, Ci and WUEi, H×I significantly affected Gs, T, Ci, WUEi, and S×H×I significantly affected Gs and Ci (**Table 2**).

**Table 2:** Photosynthetic characteristics of leaves under Pb and Zn stresses inoculated AM fungi.

Treatment	P_n_(μmol m^-2^s^-1^)	G_s_ (μmol m^-2^s^-1^)	C_i_ (μmol mol^-1^)	T (mmol m^-2^s^-1^)	WUEi
CF	9.67 ± 0.94c	0.24 ± 0.02de	316.76 ± 4.68ab	9.29 ± 1.2bcde	1.07 ± 0.11def
CM	8.91 ± 0.58c	0.40 ± 0.03a	339.37 ± 12.14a	11.45 ± 1.49bc	0.82 ± 0.12f
CFA	11.65 ± 0.84abc	0.27 ± 0.02cd	318.90 ± 0.71ab	10.11 ± 0.73bcd	1.15 ± 0.00def
CMA	10.83 ± 1.21bc	0.23 ± 0.02def	300.14 ± 10.22bc	8.46 ± 0.73cde	1.28 ± 0.09de
PbF	12.81 ± 0.6ab	0.19 ± 0.01efg	265.93 ± 4.4d	7.61 ± 0.35de	1.69 ± 0.10bc
PbM	12.76 ± 0.98ab	0.14 ± 0.01g	219.79 ± 5.66e	5.87 ± 0.34e	2.21 ± 0.23a
PbFA	11.14 ± 0.68abc	0.28 ± 0.02cd	323.78 ± 10.13ab	12.45 ± 1.52b	0.93 ± 0.11ef
PbMA	13.29 ± 0.99ab	0.27 ± 0cd	306.11 ± 6.1bc	9.74 ± 0.78bcd	1.37 ± 0.02cd
ZnF	13.9 ± 0.55ab	0.16 ± 0.02fg	254.77 ± 3.68d	7.93 ± 0.7cde	1.78 ± 0.11b
ZnM	11.59 ± 0.57abc	0.22 ± 0.01def	290.38 ± 8.18c	9.70 ± 0.67bcd	1.21 ± 0.08de
ZnFA	13.47 ± 0.71ab	0.35 ± 0.01ab	316.60 ± 2.38ab	16.37 ± 0.96a	0.83 ± 0.05f
ZnMA	14.19 ± 1.75a	0.32 ± 0.04bc	307.51 ± 13.4bc	16.52 ± 2.18a	0.92 ± 0.17ef
*S*	ns	ns	ns	ns	ns
*H*	***	***	***	***	***
*I*	ns	***	***	***	***
*S×H*	ns	*	***	ns	***
*S×I*	ns	**	*	ns	*
*I×H*	ns	***	***	***	***
*S×H×I*	ns	***	**	ns	ns

P_n_, The net photosynthetic rate; T, transpiration rate; C_i_, intercellular CO_2_ concentration; G_s_, stomatal conductance; WUEi, water use efficiency. Different letters indicate significant differences among treatments according to Duncan’s test (P< 0.05). Each value is the mean ± SE (n = 4). The meanings of abbreviations are the same as the description in **Table 1**.

**Figure 2:**
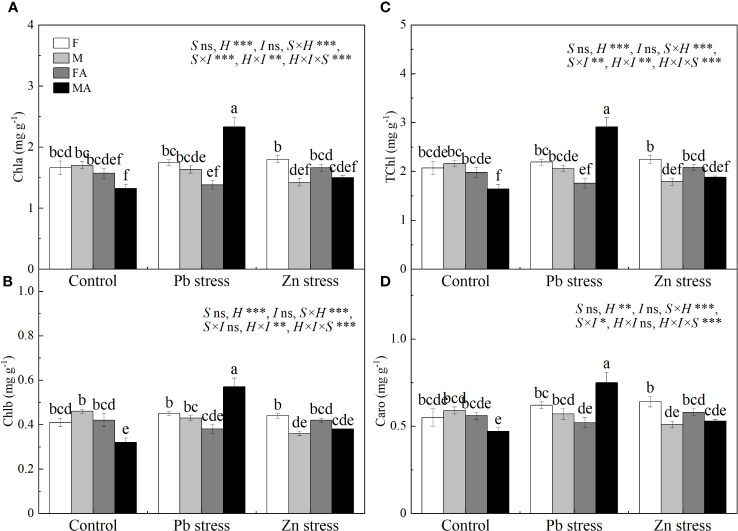
Photosynthetic pigments in *H. rhamnoides* leaves under Pb and Zn stresses inoculated AM fungi: **(A)** Chla content; **(B)** Chlb content; **(C)** TChl content; **(D)** Caro content. Different letters indicate significant differences among treatments according to Duncan’s test (P< 0.05). Each value is the mean ± SE (n = 4). F, Female; M, Male; FA, Female inoculated with AM fungi (Female + AM); MA, Male inoculated with AM fungi (Male + AM).

Compared with Pb stress, females showed lower Chla, Chlb, TChl and Caro, while males possessed higher Chla, Chlb, TChl and Caro under Pb+AM treatment (**Figure 2**). Moreover, the chlorophyll and carotenoid contents of males were higher than those of females under Pb +AM and males under Zn+AM treatment. In addition, compared with females, Zn stress decreased the contents of Chla, Chlb and TChl in males by 16.47%, 21.74% and 17.13% respectively. However, females showed higher Chla and TChl contents under Zn+AM treatment than females under Pb+AM treatment. In short, females showed lower contents of Chla, Chlb, TChl and Caro under Pb+AM treatment than those under Pb stress, while males showed an increasing trend. The interaction of S×I significantly affected Chla TChl and Caro. The interaction of H×I significantly affected Chla, Chlb and TChl. The interaction of S×H and S×H×I significantly affected Chla, Chlb, TChl and Caro (**Figure 2**).

### Effects of heavy metals and AM fungi on MDA, antioxidant enzymes and proline

**Figure 3:**
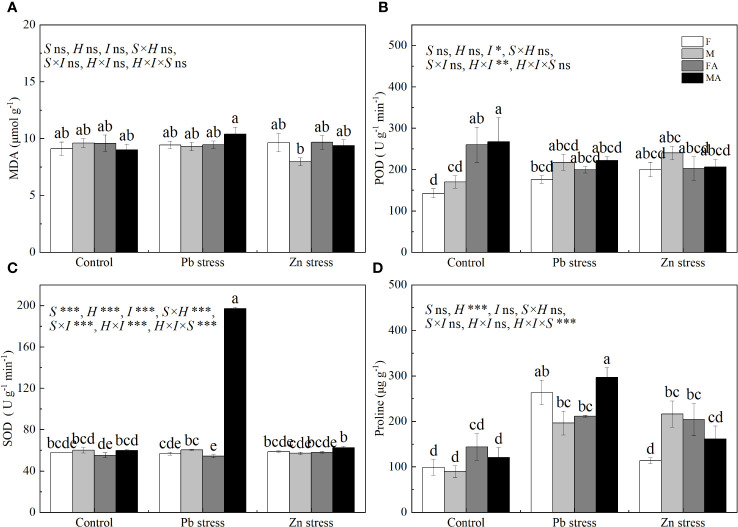
MDA, antioxidant enzymes and Pro accumulation of *H. rhamnoides* under Pb and Zn stresses inoculated AM fungi: **(A)** MDA content in leaves; **(B)** POD activity in leaves; **(C)** SOD activity in leaves; **(D)** Proline accumulation in leaves. Different letters indicate significant differences among treatments according to Duncan’s test (P< 0.05). Each value is the mean ± SE (n = 4). F, Female; M, Male; FA, Female inoculated with AM fungi (Female + AM); MA, Male inoculated with AM fungi (Male + AM).

Pb+AM and Zn+AM treatments significantly increased the SOD activity of males by 226.01% and 9.46% respectively compared with Pb and Zn stress treatments, and the SOD activity of males under Pb+AM was much higher than did males under Zn+AM (**Figure 3C**). The proline accumulation of both sexes increased significantly by 166.12% and 119.61% under Pb stress, and the Pro of males also increased significantly by 79.82% under Zn stress compared with the controls. Pb+AM treatment increased by 51.36% significantly the Pro of males that was higher than females compared with Pn stress, while Zn stress significantly increased the Pro of females (**Figure 3D**). In a short, Pb+AM treatment significantly increased SOD activity and Pro of males compared with the Pb stress, and Zn+AM significantly increased males SOD and females Pro accumulation compared with Zn stress. The interaction of Pb×I significantly affected POD and SOD, Pb×S significantly affected SOD. The interaction of S×H and S×I significantly affected SOD, H×I significantly affected POD and SOD. The interaction of S×H×I significantly affected SOD and Pro (**Figure 3**).

### Effects of heavy metals and AM fungi on the distribution of starch and soluble sugar

**Figure 4:**
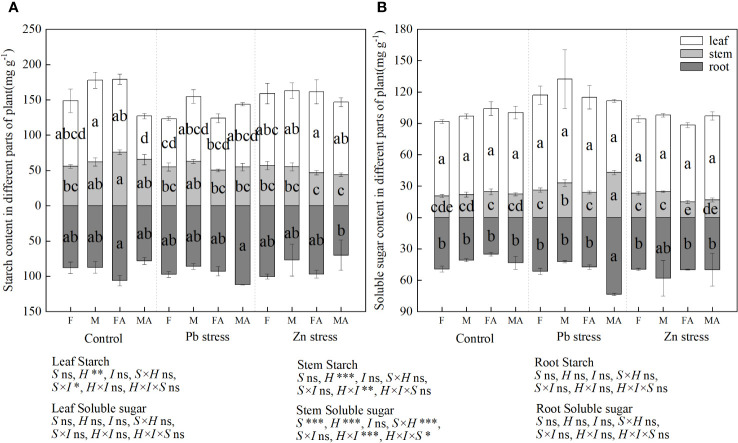
Distribution of starch and soluble sugar content of *H. rhamnoides* under Pb and Zn stresses inoculated AM fungi: **(A)** Starch content in different part of plant; **(B)** Soluble sugar content in different parts of plant. Different letters indicate significant differences among treatments according to Duncan’s test (P< 0.05). Each value is the mean ± SE (n = 4). F, Female; M, Male; FA, Female inoculated with AM fungi (Female + AM); MA, Male inoculated with AM fungi (Male + AM).

Compared with the controls, the leaf starch content of males decreased by 33.66% under CK+AM, and the stem starch content of females increased significantly by 36.38% (**Figure 4A**). However, males possessed a lower root starch content under Zn+AM than males under Pb+AM treatment, while females showed a higher leaf starch content than did females under Pb+AM treatment. Compared with the controls, males showed higher stems soluble sugar content under Pb stress and Pb+AM treatments while females showed lower stem soluble sugar content under Zn+AM (**Figure 4B**). The stems soluble sugar content of both sexes under Pb+AM were higher than did males and females under Zn+AM. In addition, the root soluble sugar content under Pb+AM was significantly increased compared with the controls and Zn treatments by 69.53% and 47.55%. The results showed that the males produced more soluble sugar under Pb stress, and Pb+AM treatment increased root soluble sugar content of males. The interaction of H×I significantly affected stem starch and stem soluble sugar, S×I significantly affected the leaf starch. The interaction of S×H and S×H×I significantly affected stem starch (**Figure 4**).

### Effects of heavy metals and AM fungi on Pb or Zn content, transfer and enrichment coefficient

**Figure 5:**
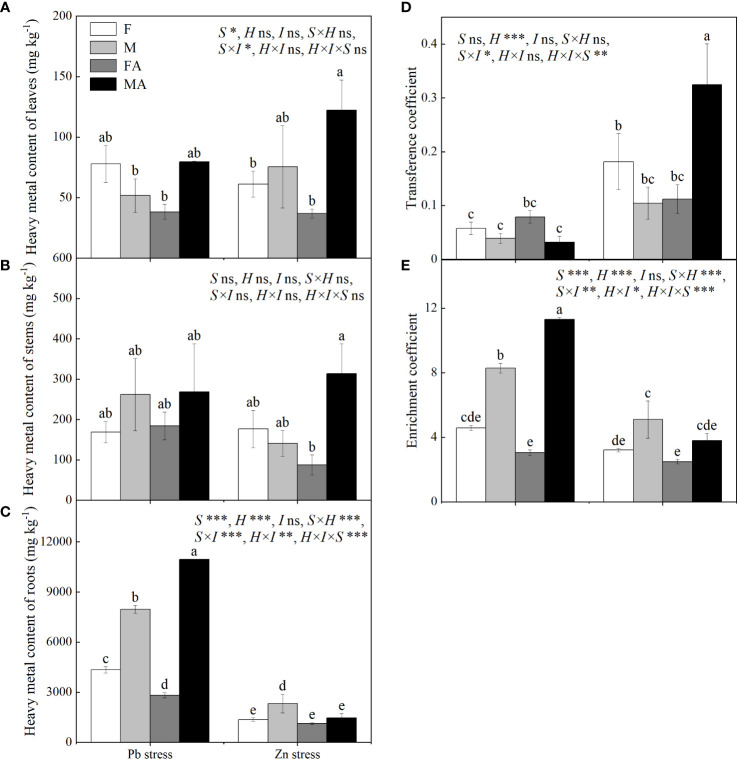
Pb and Zn contents, transfer and enrichment coefficient of *H. rhamnoides* under Pb and Zn stresses inoculated AM fungi: **(A)** Heavy metal content of leaves; **(B)** Heavy metal content of stem; **(C)** Heavy metal content of roots; **(D)** Transference coefficient; **(E)** Enrichment coefficient. Different letters indicate significant differences among treatments according to Duncan’s test (P< 0.05). Each value is the mean ± SE (n = 3). F, Female; M, Male; FA, Female inoculated with AM fungi (Female+AM); MA, Male inoculated with AM fungi (Male+AM). ns: no significant difference; *P < 0.05; **0.01≤P < 0.001; and ***P≤0.001.

Males showed higher roots heavy metals contents than did females under Pb and Zn stresses (**Figure 5C**). Pb+AM treatment significantly decreased the root Pb content of females by 34.96%, while increased root Pb content of males by 37.45% (**Figure 5C**). Compared with Zn stress, males showed higher leaf and stem Zn contents than did females under Zn+AM (**Figures 5A, B**). The root Zn content of males decreased significantly by 37.34% under Zn+AM and no significant sex-related differences were observed between females and males compared to Zn stress (**Figure 5C**). No significant differences in transfer coefficient were observed in both sexes under Pb+AM compared to Pb stress, while males showed higher transfer coefficient than did females under Zn+AM compared to Zn stress (**Figures 5D-F**). Under Pb and Zn stresses, the enrichment coefficient of males were higher than did females. The enrichment coefficient of males increased significantly by 36.45% and higher than did females under Pb+AM stress, while Zn+AM treatment had no significantly influence (**Figure 5E**). In short, the heavy metal contents of both sexes were mostly enriched in the roots, and the root Pb content and the Zn content in aboveground part of males were higher than those females under the Pb+AM and Zn+AM treatments. Pb+AM treatment increased the enrichment ability, while Zn + AM treatment improved the transfer ability and tolerance of males to Zn stress. The interaction of S×H and H×I significantly affected Pb and Zn content of roots and enrichment coefficient, S×I significantly affected Pb and Zn content of leaves and roots, transfer, enrichment coefficient, while S×H×I significantly affected Pb and Zn content of roots, transfer and enrichment coefficient (**Figure 5**).

### Principal component analysis of physiological and biochemical parameters

**Figure 6:**
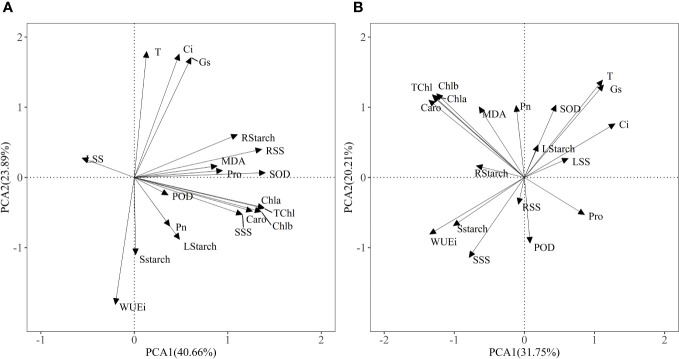
Results of PCA showing: **(A)** physiological indexes of *H. rhamnoides* under Pb stress and AM treatment; **(B)** physiological indexes of *H. rhamnoides* under Zn stress and AM treatment. Pn, The net photosynthetic rate; T, transpiration rate; Ci, intercellular CO_2_ concentration; Gs, stomatal conductance; WUEi, water use efficiency; TChl, total chlorophyll content; Chla, chlorophyll a; Chlb, chlorophyll b; Caro, carotenoid; LStarch, starch content of leaf; SStarch, starch content of stem; RStarch, starch content of root; LSS, soluble sugar content of leaf; SSS, soluble sugar content of stem; RSS, soluble sugar content of root; MDA, malondialdehyde; SOD, superoxide; POD, peroxidase; Pro, proline.

The PCA model of two principal components explained 64.55% of the physiological variance under Pb treatments (**Figure 6A**) and 51.96% of physiological variance in Zn stress and AM treatments (**Figure 6B**), indicating that Pb or Zn stress and AM treatment differences affect the physio-biochemical traits. The first component (40.66%) was strongly influenced by photosynthetic pigments, SOD, root soluble sugar and the physiological indexes were positively correlated with PC1 except WUEi under Pb stress and Pb + AM treatments. In Zn stress and Zn + AM treatments, the first component (31.75%) was strongly influenced by Ci, WUEi, Chla, Chlb, TChl, Caro, and the Chla, Chlb, TChl, Caro in Zn treatments were significantly positively correlated with PC1 while Ci and WUEi showed a significant negative correlation whit PC1.

## Discussion

### Effects of AM fungi on the growth of *H. rhamnoides* under Pb and Zn stress

Our results showed that both sexes of *H. rhamnoides* were infected by AM fungi under Pb+AM and Zn+AM treatments. Although inoculation with AM fungi significantly improved the salt tolerance, drought tolerance and resistance to heavy metal persecution of host plants (Qiu et al., 2020; Zarik et al.2016; Zhang et al., 2020), whether a significant correlation existed between infection rate and plant eco-physiological responses has not been concluded. When plants are under stress conditions, the roots receive signals and transmit them to AM fungi, and AM fungi promotes the formation of arbuscular through signal transduction, thereby increasing its infection rate, resulting in superior root morphology to enhance plant tolerance to stress (Rillig et al., 2010). However, the infection rate gradually may also decrease with the increase of stress degree (Li et al., 2019). The males of *H. rhamnoides* still maintained a high infection rate under Zn stress and reduced the infection rate of females significantly, which may be related to the sexual dimorphism of tolerance to heavy metal stress.

Our results are similar to the responses previously discovered in *Neyraudia reynaudiana* when the Pb concentration was less than 100 μM (Zhou et al., 2016). The biomass of males decreased significantly under Pb+AM and Zn+AM treatments, but the SOD activity and the accumulation of heavy metal content in males increased significantly. The mechanism of plant coordinated growth-defense balance shows that plants tend to transfer more resources to activate defense systems to resist stress at the expense of normal growth and development when they are under stresses (Li et al., 2019). It may transfer more resources to activate the defense system under Pb + AM treatment, resulting in a significant decrease in biomass during the experiment. It has been reported that under Pb stress, males of *Populus deltoides* accumulate Pb in the roots and inhibit the transport of heavy metals (Xu et al., 2016). Heavy metals content was also accumulated in the roots of *H. rhamnoides* under Pb+AM treatment, indicating that males of *H. rhamnoides* could reduce the toxicity of heavy metals by reducing biomass and increasing the absorption of heavy metals by roots.

### Effects of AM fungi on physiological and biochemical responses of *H. rhamnoides* under Pb and Zn stress

In the present study, Pb stress significantly decreased Gs, Ci and T, while Zn stress decreased Gs and Ci, increased Pn and WUEi significantly. It indicated that the protective mechanism of *H. rhamnoides* to reduce heavy metal toxicity is triggered by closing stomata to reduce CO_2_ and water absorption, so as to reduce the accumulation of heavy metals in plants. And plants will also increase WUEi by reducing Gs and T (Wang et al., 2013; Gan et al., 2018). Under Zn + AM treatment, the Gs and T of both sexes increased significantly. The opening of stomata increased Gs and absorbed more CO_2_ to enhance the photosynthetic capacity of plants. This may be a self-protective stress response of *H. rhamnoides* to adapt to the damage caused by stress, which improves the adaptability of *H. rhamnoides* to heavy metal conditions and the plasticity of photosynthetic capacity (Han et al., 2010; Chen et al., 2017). Our results showed that the photosynthetic capacity of males under Zn stress was significantly higher than did male under Pb stress and the T of both sexes under Zn + AMF treatment was significantly higher than that of Pb + AM (**Table 2**), indicating that the promotion effect of AM fungi on the photosynthetic capacity of *H. rhamnoides* under Zn stress was significantly higher than that under Pb stress. Heavy metals can affect plants photosynthesis and growth by affecting the synthesis of chlorophyll (Zhang et al., 2012), and can also be used to indicate the damage of plants tissues and organs under stresses. Our study showed that Pb + AM treatment increased the photosynthetic pigment synthesis ability of males, Zn stress inhibited the synthesis of male photosynthetic pigments, but it had no significant effect on the photosynthetic pigment synthesis ability of *H. rhamnoides* under Zn + AM treatment. It indicated that the capture of light energy of *H. rhamnoides* increased after inoculation with AM fungi, which affected the light energy utilization efficiency and plant growth of male plants, and the effect was greater on Pb stress than that on Zn stress.

The SOD activity of male *P. cathayana* and *P. tomentosa* were significantly increased after AM fungi inoculation under salt stress (Lu et al., 2014; Wu et al., 2016). Our study showed that it had no significant effect on oxidative stress in leaf under Pb and Zn stresses. However, both Pb + AM and Zn + AM treatments increased the SOD activity of males, indicating the activation of the antioxidant enzyme system of males after inoculation with AM fungi, which improved the ability to quench intracellular reactive oxygen species in order to resist heavy metals (Gill et al., 2015). Among them, the antioxidant capacity of males under Pb +AM treatment was higher than that under Zn stress (**Figure 3C**). Our results indicated that the mycorrhizal-induced antioxidant system can enhance the ability to quench reactive oxygen molecules and increased the antioxidant capacity of male plants, which implied AM treatment increased the tolerance of male plants to heavy metals.

It has been found that after inoculation with AM the Pro of male *P. cathayana* increased under drought and salt stress (Li et al., 2015), while the Pro accumulation of female was significantly increased only after inoculation with AM under salt stress and Pb stress (Wu et al., 2016), and the Pro of male was significantly higher than that of female after inoculation with AM under Pb stress (Feng et al., 2021). In the present study, females and males can produce more Pro to regulate osmotic pressure under Pb stress, while Pb + AM treatment significantly increased the Pro accumulation of males (**Figure 3D**). It indicated that the male was significantly increased Pro accumulation after inoculation with AM fungi, which can improve the tolerance of males to Pb ions. The Pro accumulation of males increased significantly under Zn stress, while Zn + AM treatment increased the Pro accumulation of females significantly. It showed that AM had more promoting effect on osmotic adjustment of females under Zn stress.

This study showed that Pb and Zn stress did not significantly affect the starch content of each part of *H. rhamnoides*. It has been reported that *Zea mays* inoculated with AM fungi have a high accumulation of soluble sugar in tissues, especially in roots, which can make mycorrhizal plants more tolerant to osmotic pressure caused by salt stress (Feng et al., 2002). In addition, the induced defense response of plants is related to the concentration of Pb and the time of exposure to Pb stress (Jiang et al., 2018). In the present study, males showed higher stem soluble sugar than females under Pb stress, and Pb+AM treatment significantly increased the content of soluble sugar in males stem and root. Inoculation of AM fungi under this concentration of Pb treatment may induce *H. rhamnoides* to produce more osmoregulatory substances to maintain normal osmotic pressure of cells *in vivo*, and the content of non-structural carbohydrates of males was significantly increased by inoculation of AM fungi. The stem soluble sugar content of females and males decreased under Zn + AM treatment. This may be due to AM fungi improve the tolerance of plants to heavy metals, and the consumption of carbohydrates in stems is greater than decomposition (Xie et al., 2022).

### Effects of AM fungi on the accumulation and translocation of Pb and Zn in *H. rhamnoides*

After heavy metal ions enter the plant roots, they will bind to proteins to form stable chelates and remain in the roots so that plants with strong tolerance generally restricted high concentrations on heavy metals in the underground part of plants (Kushwaha et al., 2018). It is generally believed that the root system is the main accumulation site of Pb (Zhou et al., 2016) and Zn, and the Zn content in the aboveground part increases with the increase of stress time (Grassi et al., 2020; Liao et al., 2022). The results of this study showed that the content of heavy metals in *H. rhamnoides* root was higher than that in aboveground parts under Pb stress and Zn stress. The reasons for this phenomenon may include immobilization or precipitation by negatively charged lignin and pectins or binding to carboxylic group of mucilage uronic acids within the cell walls (Arias et al., 2010), or in intercellular spaces (Malecka et al., 2009) or vacuolar sequestration in the cortical and rhizodermal cells (Pourrut et al., 2011). Under Pb + AM treatment, the Pb content in male roots was significantly increased, and the male had a stronger fixation effect on Pb after AM inoculation. After Zn + AM treatment, the male transport and enrichment coefficient were increased, indicating that inoculation with AM fungi improved the transport and absorption of males to Zn. It indicated that H. *rhamnoides* has high phytoremediation potential for heavy metals. The planting of male *H. rhamnoides* with AM in Pb contaminated areas is conducive to fixing Pb in the roots of males to reduce the concentration of heavy metals in the soil. On the other hand, the tolerance of *H. rhamnoides* after inoculation with AM was enhanced in the Zn contaminated area, which promoted the potential of phytoremediation by the improvement of plant growth under heavy metal stress.

## Conclusion

Our results indicated that the interaction between Pb or Zn stress and AM fungi inoculation significantly affected the accumulation and transport of heavy metals, biomass, photosynthetic capacity, antioxidant enzyme activity, osmotic adjustment substances and content of non-structural carbohydrates in males and females of *H. rhamnoides*. AM fungi treatment improved the photosynthetic capacity, osmotic adjustment ability and antioxidant capacity of males under Pb stress. AM fungi treatment had a significant effect on the osmotic adjustment ability of females, the antioxidant capacity of male, and the photosynthetic capacity of males and females under Zn stress. The root nonstructural carbohydrate production of males inoculated by AM fungi under Pb stress was significantly higher than that of males inoculated by AM fungi under Zn stress, while AM fungi treatment promoted the increase of leaf biomass of males under Zn stress. The absorption capacity of males to Pb was stronger, when inoculated with AM fungi, the transport capacity of females was stronger under Pb stress than Zn stress, while AM fungi improved the transport capacity and tolerance of males to Zn stress. In conclusion, two sexes of *H. rhamnoides* inoculated with AM fungi had significant gender dimorphism in response to different heavy metals, and inoculation with AM fungi had a beneficial effect to enhance tolerance of *H. rhamnoides*, which may improve the phytoremediation potential of dioecious *H. rhamnoides* to Pb and Zn contaminated soils. In the future, more attention should be paid to the promoting effect of AM fungi on plants, including the benefits of the improvement of tolerance. Furthermore, the sexual-specific responses and phytoremediation of dioecious plants in heavy metal polluted soils should be explored.

## Data availability statement

The raw data supporting the conclusions of this article will be made available by the authors, without undue reservation.

## Author contributions

JC: Conceptualization, Project administration, Supervision, Writing – review & editing. LF: Writing – original draft. ZZ: Investigation, Writing – review & editing. QJ: Investigation, Writing – review & editing. YL: Investigation, Writing – review & editing. HC: Data curation, Writing – review & editing. YH: Investigation, Writing – review & editing.
